# Children should be seen and also heard: an explorative qualitative study into the influences on children’s choice of footwear, their perception of comfort and the language they use to describe footwear experiences

**DOI:** 10.1186/s13047-021-00487-3

**Published:** 2021-07-16

**Authors:** Carina Price, Sue Skidmore, Jane Ratcliffe, Anita Williams

**Affiliations:** University of Salford School of Health and Society, Brian Blatchford Building, Frederick Road Campus, Greater Manchester, M6 6PU Salford, England

**Keywords:** Footwear, Comfort, Children, Qualitative, Choice

## Abstract

**Background:**

Footwear has an essential role including protection of the feet, overall performance, foot health and potentially, supporting normal development of the foot. In addition to these physical aspects which may influence choice of footwear design, there are psychological influences on what a person chooses to wear. The concept of footwear ‘comfort’ spans physical and psychological perceptions of comfort in adults. However, there is little understanding of what influences children’s footwear choices, how children perceive footwear comfort, or the language used to describe footwear experiences. Therefore, this study aimed to explore these three parameters as the first step to informing the development of a scale to measure footwear comfort in children.

**Methods:**

A pragmatic qualitative design with thematic analysis as an analytical approach was implemented. Passive observation and short interviews were carried out with 23 children (aged 1–12 years) at a footwear manufactures headquarters and store. Prompts included shoes being tried on and field-notes were taken relating to verbal and non-verbal communication. Field notes were coded then themes were identified, reviewed and named.

**Results:**

Overall, the children equated comfort to softness. However, influences on footwear choice were multidimensional including aesthetics, psychosocial influences, identified ‘comfort’ and ‘discomfort’ areas, practical issues and predictive concerns; all interacting with the age of the child.

**Conclusions:**

For children, footwear comfort is a complex phenomenon having physical, cognitive, social and emotional developmental components. This can be seen in how the children perceive the ‘feel’ of the shoe and how the shoe is assessed in the context of how the shoe meets the child’s physical and psychosocial developmental needs. In younger children footwear preference is related to idiosyncratic tastes in aesthetics, physical ability and comfort. As children age, societal influences begin to expand the social function of footwear denoting group membership, to include themes that transcend the functional and social function of footwear. The knowledge from this study can inform the development of age group specific tools to evaluate comfort.

## Background

Foot health in childhood can be influenced by footwear as children’s feet react more sensitively to external factors [1–3]. Footwear may negatively influence foot development and morphology, the influence mediated by style and fit [1, 4]. Footwear use in children influences spatio-temporal parameters of gait compared to barefoot [5, 6] with ill-fitting footwear also further influencing children’s gait [7, 8]. Shoes that are too big effect spatio-temporal parameters including reducing step length and increasing toe clearance [7]. Lower limb kinematics are also altered, notably increasing knee flexion and ankle plantar-flexion in swing [8]. Research with adult participants shows us that footwear that is too small can influence spatio-temporal parameters, specifically reducing gait velocity by reducing stride length [9]. Beyond the immediate impacts of footwear on gait, influences on foot development and morphology are apparent. Habitually barefoot populations and those wearing open shoes, have higher arches than habitually shod populations [10–12]. However, as Yurt et al. describe, footwear is essential for protection of the child’s foot and is a social convention in many societies [13]. Similarly, Morrison et al., suggest that a deeper understanding of the ‘social dimensions’ of footwear worn by children is required, such as the influence of fashion-trends and branding as children age and become more influential in decision making relating to footwear purchasing [14]. Of particular interest is how these factors may influence a child’s perception of comfort and desire to wear appropriate footwear which is crucial to ensuring that comfort is measured accounting for these ‘social dimensions’.

Most investigations into influences on footwear choices and comfort have been undertaken in adults [15, 16], with a variety of methods for capturing comfort such as Visual Analogue Scales [17], Likert Scales [18] and ranking of footwear by preference [19]. The scale utilised to quantify comfort is also likely to influence the result [19], with a less complex scale being more reliable [20] and therefore careful selection of tools and language is required. Visual analogue scales are commonplace; however they rely on appropriate anchor words for the footwear style and user. Some authors blind wearers to the shoe aesthetics in an attempt to prevent them from influencing subjective comfort [21] and other researchers include aesthetics aspects in their data collection [18].

Understanding footwear comfort in children is important for the manufacturer, researcher or clinician trying to provide, and measure, a comfortable footwear experience. In order to be able to quantify comfort in children we need a tool that is specific to their cognitive, social and emotional developmental stage as well as their physical development. In children the foot is developing, with softer tissues, varying relative dimensions and requirements for rapid growth [3]. Cognitive, social and emotional development is also rapid, including language comprehension and the child establishing self-identify. Therefore, to develop an effective tool, firstly we need to understand what influences footwear choices in children. Secondly, what influences footwear comfort perception from physical to cognitive, social and emotional factors. Thirdly, the definition of an appropriate tool with anchor words which are age appropriate is also key. Before this can be achieved there needs to be an understanding of the language that children use relating to footwear to be able to develop a tool to capture levels of comfort. Hence this study aims to describe the language children use to describe their footwear and footwear experiences and explore influences on their footwear choices.

## Methods

 This qualitative study received ethical approval from the University of Salford (HST1920-175).

### Participants

A convenience sample of children was recruited at a footwear manufacturer’s fitting session, or in the same manufacturers store. Inclusion criteria were that the participant and parent/guardian was consenting; the child could ambulate, had no medically- reported issues with pathological foot pain and the ability to express themselves in age appropriate English language and in order to understand and respond to the questions. Parent/guardians were approached in-person, given a Participant Information sheet detailing the research and the data collection process was described to both the parent/guardian and child. This described the aims of the research and the contribution of the research topic to the researcher’s qualification. Written consent was obtained from parent/guardian on behalf of themselves and their child and children verbally consented and children were accompanied throughout. No participants and parents who were approached refused to participate or dropped out once participating.

### Methodology

Data collection took place using a pragmatic qualitative approach to both collection and analysis. A pragmatic approach was adopted because it focuses on research in the experiential context appropriate to the research question [22–24]. The relevance of the setting is particularly important when conducting research with children [25, 26] and therefore pragmatically this real-world encounter was deemed the most appropriate context to engage children within, while they were both physically and cognitively engaged in the research subject matter within a shoe shop.

### Data collection

A researcher (JR- Female, MSc Podiatry final year, with experience of working with children) passively observed children trying on shoes and, with field notes, recorded words used by the participants to talk about footwear and fit and any terms used to refer to their feet or shoes. Non-verbal communication was also recorded within the field notes, describing the action and the context in which it was made. Non-verbal communication was defined as facial expression and physical gestures such as nodding of the head and pointing at areas of the foot. Comments from parent/guardians were recorded alongside those from the children. After passive observation the researcher conducted a short interview (approximately 5 min) with each child to explore the influences on footwear choice and comfort. Example questions included *“What words might you use to describe how a pair of shoes feels?”* and *“What else matters to you when buying shoes?”*. Prompts were used such as the shoes being tried on, the children’s own shoes and diagrams of shoes and feet. No repeat interviews were conducted, and transcripts were not returned to participants for comment.

### Data analysis

Data analysis followed the six-step thematic analysis framework [27]. All field notes were read, and codes were developed. Initial codes were then collated to gather data into themes and the frequency of codes was used as a mechanism to facilitate this considering the short phrases and responses from the children [28]. Coded extracts were then reviewed within their themes and subsequently defined and named [27]. Codes and themes for the verbal and non-verbal communication for both parent/guardian and child were confirmed with second (SS) and third (AW) researchers and not reviewed by participants. Children were grouped by age in relation to development in the context of society and stages of psychosocial development [29]. Narrative extracts from interviews and observations are presented to convey the nature of the language used and support findings.

## Results

Twenty-three children (13 Female; 1–3 years (*n* = 2); 4–6 years (*n* = 10) and 7–12 years (*n* = 11)) participated in the study detailed and identified in Table 1.

**Table 1:** Participant demographics and number for reference and attribution of quotations

Participant Number	Gender	Age (years)	Age group
**P01**	**F**	**6**	**4–6**
**P02**	**M**	**11**	**7–12**
**P03**	**F**	**9**	**7–12**
**P04**	**M**	**5**	**4–6**
**P05**	**F**	**8**	**7–12**
**P06**	**F**	**10**	**7–12**
**P07**	**M**	**8**	**7–12**
**P08**	**M**	**11**	**7–12**
**P09**	**F**	**10**	**7–12**
**P10**	**F**	**6**	**4–6**
**P11**	**F**	**6**	**4–6**
**P12**	**F**	**5**	**4–6**
**P13**	**F**	**6**	**4–6**
**P14**	**F**	**3**	**1–3**
**P15**	**M**	**3**	**1–3**
**P16**	**M**	**5**	**4–6**
**P17**	**F**	**5**	**4–6**
**P18**	**M**	**12**	**7–12**
**P19**	**F**	**10**	**7–12**
**P20**	**F**	**6**	**4–6**
**P21**	**M**	**11**	**7–12**
**P22**	**M**	**10**	**7–12**
**P23**	**M**	**6**	**4–6**

 Thirty-one codes were identified from non-verbal communication (observed) and verbal communication (with parent/guardian input), which were then organised into five themes:

1. Aesthetics of footwear; The indication of visual (un)appeal.
2. Psychosocial influences on footwear choice; Social influence on thought process and behaviour.
3. Footwear comfort/discomfort; Aspects that make it physically pleasant or unpleasant to wear.
4. Practical issues with footwear; Indication of a specific functional requirement.
5. Predictive concerns about footwear; Indication that an issue may occur in the future.

The multi-faceted narrative nature of the data meant that codes often came under more than one theme. This was particularly in themes 3,4 and 5, which were found to relate mainly to older children.

### Theme 1– Aesthetics of footwear

Visual appeal was commonly the first description of the footwear from children:

*‘I want lizard shoes. I like them’ (age 3, P14)* and *‘I like the diamonds’ (age 6, P11*.

Dissatisfaction with the visuals of a shoe was also identified:

*‘Don’t like aeroplanes’ (age 5, P04)*.

The initial comments about footwear in the younger group are restricted to a single consideration:

*‘Good, nice’ (age 5, P05), ‘Cute’ (age 6, P13), ‘Urggh’ (age 6, P13)* and *‘Fine, shiny’ (age 6, P20).*

More complex ideas start to be expressed in the data regarding physical aesthetics from 8 years of age onwards, in addition to referencing what is physically in front of them:

*‘Evil. They are kind of high heeled. That (points to sole) is an odd shape…. I don’t like pink’ (age 9, P03*).

The older children started to expand from a one-dimensional representation of aesthetic appeal to incorporate underlying concepts such as footwear fit, shape and colour:

*‘I don’t like long ones (shoes with a point… Don’t like pointy shoes) (age 11, P02).*

One child offers a reason why shoes are liked and goes on to say that she wants them despite them feeling loose:

*‘They feel floppy. They have jewels and hearts I want to get them anyway.’ (age 8, P05).*

The overall shape of the shoe is also regularly commented on and children judged aesthetics of the shoe on a belief that it would be uncomfortable based on prior experience:

‘*Wedged in would make it uncomfortable*……. *I don’t like the point’ (age 11, P02)*.

The older children in the study also used their experiences of observations of adults:

*‘My dad’s shoes look stupid, they are grandad shoes’ (age 12, P18)*.

This is also indicative of a growing social awareness.

 Non-verbal communication was evident across all age groups of children with facial expressions supporting their opinion such as frowning (age 6, P13), disgusted faces (age 11, P21), head shaking (aged 10, P19), and kissing to indicate approval (age 6, P10).

Parent/guardian input included comments expressing parental feelings of conflict about compromise between comfort, fit, aesthetics and choice:

*‘She likes ballet pumps….but will complain they aren’t comfortable’(parent of child aged 5, P17)*.

With another comment from a grandparent relating to aesthetic choice:

*‘Not enough new choice since September, kids want something different’ (Grandma of child age 6, P09).*

Aesthetics is one of the first considerations for children, with the older children demonstrating more complex narrative. Parent/guardian are aware of the balance between aesthetics, fit and comfort.

### Theme 2- Psychological influences on footwear choice

The ownership of a product, in this case footwear, influences the way the child sees themselves. Expressing self as part of a group through product ownership is also congruent with convention: *‘These won’t look nice with dresses’ (age 10, P06).*

A sense of belonging to a group was expressed specifically to an age group. Being perceived as too young may have connotations of dependency: ‘*Hideous, Velcro is for babies’ (age 10, P19*). Also being perceived as old-fashioned was evident: ‘*Granny shoes’ (age 10, P06).*

Footwear allows a child to express themselves as part of a group: *‘My friend has them, I like them’ (age 5, P04).* It also enables them to express individuality through differentiating themselves: ‘*I want different from my sister’ (age 6, P10)*.

Parental comments were related to the importance of children’s individuality:

*‘My son likes to stand out’ (parent of child age 11, P21)* and *‘Not enough new choice since September, kids want something different’ (grandma of child age 10, P09)*.

Parent/guardian comments expresses the problems and frustration that caregivers feel through lack of choice in footwear and on some level acknowledges the part that footwear plays in facilitating the development of self-identity and autonomy. This is also referenced in terms of belonging to a peer group, particularly in the school-aged children:

*‘She wants what her friend has’ (parent of child age 10, P19)* and *‘Too much glitter, she only liked glitter when she started school’ (parent of child age 6, P01).*

There was also confirmation of opinions being reinforced by older siblings (*parent of child age 3, P15*).

This theme identifies the power of footwear in relation to identity and belonging to a specific age or peer group.

### Theme 3 – Footwear comfort/discomfort

The youngest age group referred to the footwear generally with reference to comfort: *‘These are comfy, good’ (age 3, P14)* and discomfort was related to lack of softness *‘They feel hard’ (age 3 P15).* This shows that younger children were able to identify footwear preference relating to comfort and discomfort.

For both older groups softness and accommodating: *‘I can wiggle my toes’ (age 6, P20)*, ‘*There is lots of soft (pointing at padding)’ (age 10, P22)* and *‘Not hard around the side’ (age 5, P12)* were revealed as the main factors that maximised perceptions of comfort. Again, tightness or not accommodating the foot was associated with discomfort: ‘*Feels tight’ (age 5, P16), ‘Too little’ (age 6, P11) and ‘Too tight’ (age 6, P13).*

Shoes being too large was also associated with discomfort: *‘They are a bit big at the back’ (age 10, P19)* and *‘They are loose’ (age 6, P20)*. However, this was not as frequent as comments relating to footwear being too small or too tight.

The oldest age group also identified inflexibility in addition to the softness and fit aspects identified in the younger children: *‘rather have a thick sole, but won’t bend’ (age 10 P06).*

Older children related more specific areas of footwear to comfort, particularly the heel rubbing, slipping or being too hard, *‘They slip’ (age 9, P03) and ‘Harder on the back’ (age 10, P19).* They also identified the sole as influential to comfort, *‘Sole feels good, I don’t normally notice anywhere except the sole’ (age 9, P03)* and *‘I don’t like hard soles, they are heavy’ (age 12, P18).*

There were also indications that the toe area influenced comfort. A child pointed at the base of left 5th metatarsal indicating pain (age 6, P13) and others pointed to the toes to indicate discomfort (age 11, P02; age 10, P22).

Another footwear feature which was specifically referred to was the upper:

‘*They are hard, I like shoes that are softish and soft tongue’ (age 11, P02)* and ‘*That bit not comfy (points to tongue) it irritates me’ (age 10, P06).*

One child expressed specific knowledge of how inappropriate ‘fit’ of footwear can result in a problem with comfort, ‘*Length is important, too much room across the top is not good as can crease and dig in’ (age 12, P18).*

For both 4–6 and 7–12 age groups the width of the footwear was a key issue when perceiving comfort: ‘*Comfy, my width is D’ (age 10, P06), ‘Width wrong’ (age 11, P21)* and *‘Sides are too wide’ (age 5).*

*Non-verbal communication was also used to show things were too loose, such as pointing at the dorsum where the upper was loose (aged 11, P08).*

In addition to specific footwear features which enable comfort, the psychosocial aspects of comfort start to appear in children, with the focus being on it as a facilitator. In the youngest children this was demonstrated through performance of physical skills such as dancing (aged 3, P14) and running (aged 3, P15; aged 6 P23). In the older children this was verbally described: *‘I can run’ (age 5, P16)* and *‘It’s good when I jump’ (age 5, P17).*

From age 9 onwards, the comments are more complex relating to facilitation of physical skills:

*‘I like trainers, more able to do stuff, they seem good for running’ (age 9, P03)* and *‘I prefer trainers, I can move about in them more, I slide around in hard soles…no good for running’ (age 12, P18).*

Parent/guardian references to comfort were minimal however there were specific footwear styles that they associated with being uncomfortable, particularly ballet pumps: *‘She likes ballet pumps but will…complain they aren’t comfortable’ (parent of child age 5, P17).*

This theme reveals that for some, comfort equates to softness. Older children could identify areas of the footwear that contributed to comfort and discomfort including the toes, heel and sole. They also articulated what was facilitated by comfort meaning that comfort is more than a sensation, it is a facilitator and as such valued by the children.

### Theme 4- Practical issues

Relating to practicality, the majority of comments made by children related to the shoe fastening. The younger group referred to fastening in terms of it being their ability to use it:

*‘I like Velcro not buckles’ (age 6, P13) and ‘Mummy will have to tie these’ (age 6, P10)*.

The older children referred to the inability to navigate the putting on and off/fastening the shoe: *‘I struggled with the straps’ (age 9, P03).*

Some issues related to either a fit or design feature that the child felt frustrated their ability to act autonomously, *‘I can’t get them off easily’ (age 10, P19) and ‘Laces way too short’ (age 10, P06).*

There were two instances when children mentioned cost, both in older children:

*‘These are £33.60 and use to be £60 (age 8, P05) and ‘These are £13’ (age 10, P19).*

Practical issues were the focus of parents/guardians with robustness mentioned by many as a priority:

*‘Activity in school kills the shoes’, (parent of child aged 12, P18)* and *‘Priority is robustness’ (parent of child aged 5, P17).*

With shoes described as not lasting long enough. Parents also commented on the importance of suitability relating to purpose:

‘*He likes trainers as they are more comfortable, but he can’t for school. This is why he picked these; he gets heel pain’ (parent of child aged 11, P08)* and *‘He can have what he wants but must be suitable’ (parent of child aged 3, P15).*

The development of the child’s independence and autonomy was important to parents/guardians, especially in younger children and likewise to the children’s comments this related to fastening:

‘*Velcro is a priority so he can do his own shoes’ (parent of child aged 5, P04) and ‘Lace ups for younger children are not practical’ (Grandma of child aged 6, P10).*

Parent/guardian appeared to see their role as helping the child to select a shoe which was suitable for intended purpose, but also recognise the importance of autonomy. This is a balance between their role in socializing children into choices that they believe are ‘good’ for them whilst encouraging autonomy.

### Theme 5- Predictive concerns about footwear

Potential future concerns of children was noted as a distinct phenomenon as it appears to be an ability that older children possess that allows them to use their experience to predict how the shoe will meet their required needs, reflecting findings on shoe design, practicality and aesthetics. The shape of the toe box was identified as a possible issue:

*‘Wedged in would make it uncomfortable’ (age 11, P02)* and.

*‘I don’t like the point, if I was walking my toes would be forced into the end’ (age 11, P02).*

Increased risk of slipping, rubbing and pressing were also predicted from shoe fit or design:

‘*’Slip off the heel so these will slip’ (therefore rub) (age 10, P09)* and.

*‘……too much room across the top is not good as can crease and dig in’ (age 12, P18)*.

Shoe practicality and being waterproof were identified as a predictive need, for example,

*‘This will happen when I’m wearing them’ (indicates wet feet) (age 10, P06).*

Prediction that shoes will wear aesthetically well is limited to older children when assessing a shoe: *‘I like shininess but patent shoes will flake’ (age 10, P06).*

Parent/guardian input relating to prediction was related to *comfort or robustness becoming an issue with wear: ‘if they are too big they are going to rub’ (parent of child age 5, P17)* with the same parent also identifying that ballerina shoes *‘wouldn’t last 5 minutes (parent of child age 5, P17)’.*

Satisfying predictive needs about footwear, particularly comfort and aesthetic wear, is a requirement that footwear needs to overcome. This was more notable in older children and aligns with developmental theory.

## Discussion

This pragmatic study has achieved its aims of exploring what influences footwear choices in these children and providing information relating to their perception of comfort and language used to describe footwear. It has also revealed differences in relation to specific age groups. Whilst providing essential information as a first step to developing a comfort measurement tool, it is clear from this study (and with alignment to developmental theory) that any measurement tool would need to be age group specific. Overall, all children in the study equated comfort to softness and use this word consistently. However, influence on footwear choice is revealed as a multidimensional concept influenced by aesthetics, identified ‘discomfort’ in specific areas of footwear, practical issues and psychological factors. The value of the parent or guardian as an influence is also apparent, largely facilitating autonomy and choice.

The results of this study make it clear that physical comfort may not be the defining factor as to whether children chose and wear specific footwear. Seferin et al., stated that historically, footwear quickly moved from merely foot protection and assumed ‘new functions’ of aesthetic and symbolic roles [30]. Further, Nicholls et al., argue that shoes are not an inanimate object and hence play a major role in how adults perceive themselves and their identity [31]. The authors identify the need to evaluate how footwear ‘fits’ not just physically, but how footwear ‘fits’ the psychological and social needs of a wearer. This movement away from biomechanical and anthropometric aspects requires methods to assess footwear such as subjective experience and social factors.

In this study, children of all ages demonstrated aesthetic preference. In younger children this was idiosyncratic without reasoning why. Although these idiosyncratic preferences remained in older children, they developed greater complexity with conflicts between appeal, comfort and fit or practical issues. Until the age of 8 years, children were primarily concerned with physical aesthetics, after 8 years they started to give reasons why they liked some shoes, which related to inferences from aesthetics to comfort, fit or social fit. Older children also started to specifically reference what was physically in front of them. This can be linked to Theory of Aesthetic Development, which predicts pre-operational and operational stages of development where the individual moves from ‘see-like’ to ‘see and say why they like’ [32, 33]. As children move into the operational stage they link ‘I like’ with ‘why’, judging aesthetics with ego centrism, visual reasoning, emotional involvement and external influences [33]. This enables us to relate specific footwear features to their aesthetic preferences.

Older children have more complex reasoning in relation to the appraisal of the aesthetics of footwear which is judged considering perceived positive and desirable qualities. These ‘positive qualities’ become personal to the individual, although they are often linked to what an individual considers important, which in turn is linked to social influences and activities. Activities such as running, jumping and playing underpin the development of motor skills [34] as well as being important for social development and inclusion [35]. The findings of this study give some indication that facilitation of activity through footwear comfort is key. It also appears intrinsic to Erikson’s psychosocial stages of development, where children are enabled to become autonomous through motor development and facilitation of participation with others, such as through taking part in specific activities [29]. This offers some insight as to why facilitation may be so important to children when judging the appeal of footwear.

Children attending the shoe fittings within this study were primarily there to buy school shoes. Hence the influence on choice appears to be driven by parents and the need for ‘healthy footwear’ that is comfortable and reduces risk of injury. However, what constitutes a ‘healthy’ shoe for children is unclear [14], as is how shoes that are not optimized for comfort influence activity patterns [36]. For problems with comfort, both age groups identified lack of softness and not being accommodating as the main issue. This is in line with Herbaut et al., [37], whose study concluded that children perceive restriction to a greater degree than adults. It was noted during observations that, although the children’s feet were measured and the shoe size appropriate, they often complained of restriction, possibly due to firmness of materials. Children over 8 years referred to width directly, whereas those below 8 discussed it in terms of problem with shoe function such as rubbing or tightness. There appears a tolerance for comfort in relation to width, where a shoe that is too wide may be described as ‘floppy’, which may relate to material rather than dimensions. This raises the question as to how materials interact with dimensions of fit to optimize comfort.

In relation to psychological influences on footwear choice, the participants’ indicated belonging to a group defined by being feminine or masculine, casual or formal, young or old, independent or dependant, sporty or non-sporty with children looking to express this through footwear. This reflects the psychosocial stages of development from assertion of own choice to awareness of self-amongst peers [29] and may be a further component of what makes it ‘holistically’ comfortable. Being independent versus dependant is important developmentally [38]. The rejection of Velcro by the child being a rejection of outward signs of dependency rather than group belonging. Being perceived as sporty could denote Erikson’s industry versus inferiority where a child develops a sense of self in relation to peers and uses choice (in footwear for example) to facilitate this [29]. This appears to be an extension of developing physical ability and illustrates how this seems to remain important for the child, showing the transition of how developing motor skills are not a separate entity from more psychological stages but merge with it [39]. It also adds credence to the idea that there exists a unique stage in childhood that facilitation of motor activity is innately driven, which then combines with more advanced psychosocial stages and these developmental stages influence footwear choice and perception. From this study this combination is estimated to begin at around 8 years old and continue until ‘social identity’ takes greater priority leading up to adolescence [40]. Parental comment expresses the problems and frustration that caregivers feels through lack of choice in footwear and on some level acknowledges the part that footwear plays in facilitating the development of self-identity and autonomy for their child.

Potential future concerns of children was noted as a distinct phenomenon as it appears to be an ability that older children possess that allows them to use their experience to predict how the shoe will meet their required needs, reflecting findings on shoe design, practicality and aesthetics. The older children needed to be confident that footwear will have comfortable features and aesthetic aspects that are current and don’t degrade with time. The shape of the toe box was commonly identified as a possible issue and future concerns centred on fit and wear. This theme is important in relation to cognitive development. Kuhn [41] describes how early reasoning that infants display links an event to its cause and is a prerequisite for the development of prediction. This precedes the ability to make a prediction, which appears in the older children to determine if a shoe would be suitable. For this reason, it was identified as a potentially important factor in the way shoe comfort was judged, albeit with older children. Legare [42] states that young children remain centred on explanation rather than prediction, reflecting their priority of informing, constructing and revising their conceptual models of the world. This explains why the younger children did not predict any long-term use of the footwear in question, judging appeal on purely that point in time. In contrast older children’s experiences and prediction can influence perception of comfort in the present.

The influence of parents on their children was evident. This could be evidence in younger children repeating their parent’s statements or parents directing children to specific footwear requirements such as colour for example. There was no obvious evidence of conflict between parent and child however the priorities of the two did seem to differ, robustness for example was important for parent/guardians, but not addressed specifically by children independent of aesthetics. Although not directly explored in this research. it could be assumed that a carer’s priority was well-fitting footwear to optimise foot health and development [43] and children appeared to accept this. However, parent/guardians also understood the importance of choice, development of autonomy and their children expressing individuality. There appeared to be an innate ‘negotiation’ between parent/guardians and children. Negotiation in families, where children participate in consumer activities, reflects cultural ideals of independence and autonomy and have long been recognized in western consumer patterns [38]. Children have also been shown to respect these parental priorities, accepting parental ‘healthy’ advice and incorporating this into their own decisions [44], highlighting the importance of healthy footwear beliefs in carers.

### Limitations

In addition to small sample size the narrow customer profile in one shop in one location offered a limited cross section of children, which limits the generalisability of data to wider socio-economic groups and locations. In particular it may have been beneficial to capture a broader ethno-socio-economic profile with the diversity of the sample being limited due to the rural location in the South West of England and the recruitment of participants who attended as opposed to by invite. The methodology employed led to the data being the child with the influence of the parent, firstly due to the setting being a supervised one and secondly due to the data analysis methods employed.

### Future work

Non-verbal communication was evident across all age groups, warranting further investigation and development within a tool for shoe fitters. A theoretical framework relating to non-verbal communication as described by Friedman could be used to develop an understanding of how children react to footwear and conceptualize comfort [45]. This would prove valuable in understanding a child’s reaction to product and identifying their needs in a retail context [46]. Results of this study can be utilised to underpin future work aiming to develop methods of measuring footwear comfort for different age groups.

## Conclusions

This study has revealed differences in influences on footwear choice, comfort and the language used to describe both between young and older children. In older children, comfort is more complex and influenced by their footwear choices related to aesthetics, practicalities, predictive social membership and psychosocial factors. Younger children tend to have idiosyncratic tastes in beauty, ability enhancement and general comfort. These align to cognitive, social, emotional and physical stages of development and require consideration for development of comfort measurement tools.

## Data Availability

The datasets used and/or analysed during the current study are available from the corresponding author on reasonable request.
